# Immune Checkpoint Inhibitor-Induced Gastrointestinal Toxicity: The Opinion of a Gastroenterologist

**DOI:** 10.7759/cureus.19945

**Published:** 2021-11-27

**Authors:** Anca Macovei Oprescu, Raluca Tulin, Iulian Slavu, Dana Paula Venter, Constantin Oprescu

**Affiliations:** 1 Gastroenterology, Agrippa Ionescu Emergency Clinical Hospital, Bucharest, ROU; 2 Gastroenterology, Carol Davila University of Medicine and Pharmacy, Bucharest, ROU; 3 Anatomy and Embryology, Carol Davila University of Medicine and Pharmacy, Bucharest, ROU; 4 Endocrinology, Agrippa Ionescu Emergency Clinical Hospital, Bucharest, ROU; 5 General Surgery, Agrippa Ionescu Emergency Clinical Hospital, Bucharest, ROU; 6 Pediatric Surgery, Grigore Alexandrescu Emergency Pediatric Hospital, Bucharest, ROU; 7 Surgery, Bucharest Emergency Hospital, Bucharest, ROU

**Keywords:** pancreatitis, hepatitis, colitis, gastrointestinal, toxicity, immune-checkpoint inhibitors

## Abstract

Immune checkpoint inhibitors (ICIs) are currently an important component of the standard first-line treatment for many neoplasms. Some guidelines recommend ICIs as adjuvant treatment. With their increased use, the incidence of associated immune-mediated adverse reactions will also increase. A significant proportion of these reactions is represented by immune-mediated diarrhea or colitis, hepatitis, and immune-mediated pancreatic damage. The present review aims to highlight the new trends related to the diagnosis and treatment of these adverse effects depending on their degree, from the perspective of the gastroenterologist. To accomplish this, a literature search was performed, and 30 publications were considered relevant (according to the Population, Intervention, Comparison, Outcomes, and Study [PICOS] criteria). The information about each of the three toxicities in this paper was structured in two categories such as differential diagnosis and treatment. This review aims not only to increase awareness of these side effects in the gastroenterology community but also to promote the development of new treatment guidelines with contributions from gastroenterologists.

## Introduction and background

Immune checkpoint inhibitors (ICIs), which are most often represented by cytotoxic T-lymphocyte-associated protein 4 (CTLA-4) inhibitors, programmed cell death protein-1 (PD-1) inhibitors, and programmed cell death protein-1 ligand (PD-L1) inhibitors, have special importance in oncological treatment. They can be administrated alone or in combination with another ICI, with immunotherapy, and even with radiation therapy. Their mechanism of action is that they make tumor cells visible to the immune system, which can lead to immune-mediated adverse reactions. Any organ can be affected by an immune-mediated reaction, but the most common is the skin and digestive system [1].

Among the digestive system side effects, the most common are those involving the colon and small intestine, liver, and pancreas, in this order. The initial symptoms in these reactions are nonspecific and can be easily confused with the symptoms derived from the underlying disease or with toxicities of other oncological treatments [2].

These toxicities often have a low degree of severity and require only careful monitoring, and thus, discontinuation of cancer treatment is unnecessary. If, however, severe gastrointestinal, hepatic, or pancreatic reactions occur, the intervention of a gastroenterologist is recommended. These effects may lead to functional and nutritional impairment of the patient and long-term hospitalization. The specialized intervention can shorten the duration of toxicity and allow the administration of systemic oncological treatment [3]. This review contains data on three immune-induced adverse reactions (diarrhea or colitis, hepatitis, and pancreatic involvement) presented from the perspective of the gastroenterologist with the desire to raise awareness about these effects in the gastroenterology community.

## Review

A literature search was performed using the PubMed database, and the following keywords were used as search terms: gastrointestinal, immune-related adverse effects, ICIs, and toxicity. The search returned 2469 articles, of which 30 were selected as references for this review. For this selection, the Population, Intervention, Comparison, Outcomes, and Study (PICOS) criteria were used. Only articles published after 2012 were considered relevant. Original articles and meta-analyses were admitted first, but official guidelines for the treatment of these adverse reactions (European Society for Medical Oncology and National Comprehensive Cancer Network) and review publications were also consulted. The three side effects were described in separate sections, wherein the differential diagnosis and treatment options according to grade were detailed. The entire description was made from the perspective of the gastroenterologist to familiarize physicians in this medical specialty with an increasingly common reality that concerns both gastroenterologists and their fellow oncologists.

Incidence of gastroenterological ICI-induced adverse reactions

Gastrointestinal side effects, which typically occur six to eight weeks after the initiation of ICI treatment, rank second among the most common toxicities. They most often occur after combination treatment with cytotoxic T lymphocyte antigen-4 (anti-CTLA-4) and programmed death-1/programmed cell death ligand-1 (PD-1/PD-L1) inhibitors, followed in frequency by anti-CTLA-4 alone and PD-1/PD-L1 inhibitors alone. Common symptoms include watery diarrhea, sphincter incontinence, and abdominal pain [4,5].

One of the most relevant publications on the incidence of these side effects is a meta-analysis that included 8863 patients with solid tumors, who were treated with various ICIs, both in combination and alone. Most reactions were identified in patients receiving ipilimumab and nivolumab: 9.4% experienced severe colitis, and 9.2% experienced severe diarrhea. In those treated with ipilimumab alone, severe colitis and severe diarrhea had an incidence of 6.8% and 7.9%, respectively. The lowest proportion was recorded with PD-1/PD-L1 inhibitor treatment (0.9% for severe colitis and 1.2% for severe diarrhea). The incidence of these toxicities was not significantly different for various cancer sites [6].

Hepatic toxicity in ICI treatment is usually low, but some fatal cases have been reported. This effect occurs less frequently than colitis. Asymptomatic increases in aspartate transaminase and alanine transaminase are most common, and these occur in ipilimumab treatment (up to 9%) most frequently, whereas an incidence higher than 1.8% has not been reported with PD-1/PD-L1 inhibitors [7,8].

ICI-induced pancreatitis is a rare side effect. Grade 3 or 4 pancreatitis has been reported in only 1% of patients receiving ICIs. Elevated lipase levels (immune-induced growth) were described in 2.3% of patients, and the incidence of immune-induced pancreatitis was 0.3% in a major study [9].

Etiology of adverse reactions and general principles of treatment

The immune response against tumor cells is a physiological process that is based on the recognition of tumor-associated antigens (TAAs). These antigens are often similar to proteins on the surface of normal cells, and thus, the intensity of the immune response needed to eradicate tumor cells must be high. This process is also very complex. As ICIs function in the recognition of these antigens, immune-mediated adverse reactions can be triggered simultaneously, i.e., they can practically cause autoimmune damage to any organ. To activate T lymphocytes, the presentation of the antigen in the context of the major histocompatibility complex and co-stimulation through the CD28 receptor is needed [10].

The proliferation of T lymphocytes and their migration to the site of tumor cells, where they can bind to TAAs, depend on CD28. During this activation, CTLA-4 acts as a brake and competes with CD28 for binding ligands. CTLA-4 is, therefore, essential in preventing autoimmunity and maintaining tolerance to self-antigens. PD-1 is also an important checkpoint, with a structure similar to CTLA-4 but with an affinity for other ligands. The PD-1 ligand is expressed on both tumor cells and cells in the tumor microenvironment, and its action depends on inflammatory stimuli, such as interferon-gamma [11-13].

It must be understood from the beginning that if managed correctly, the majority of immune-mediated side effects are reversible. However, physicians must follow several general principles when confronted with these side effects. These principles are hit fast (experienced staff must evaluate patients from the beginning; in this regard, patients are issued brochures that briefly describe ICI treatment and can be handed to any emergency physician if needed), hit early (do not hesitate to initiate immunosuppression), hit strong (will escalate rapidly if needed), and hit short (will de-escalate treatment as soon as the patient is clinically and biologically suitable) [14].

Before detailing the treatment of these side effects, toxicity should be graded, as shown in Table 1.

**Table 1:** Standard grading in ICI-induced toxicity as reported by the Common Terminology Criteria for Adverse Events version 5.0 and the National Comprehensive Cancer Network guidelines [15,16] ADL, Activities of daily living; ULN, upper limit of normal; ICI, immune checkpoint inhibitors.

Toxicity	Grade 1	Grade 2	Grade 3	Grade 4	Grade 5
Diarrhea	Increase of fewer than four stools per day over baseline or mild increase in ostomy output compared with that at baseline	Increase of four to six stools per day over baseline or moderate increase in ostomy output compared with that at baseline; limitation of instrumental ADL	Increase of more than seven stools over baseline or severe increase in ostomy output; self-care ADL affected	Life-threatening consequences; immediate intervention required	Death
Enterocolitis	Asymptomatic; clinical or diagnostic observations only; intervention not indicated	Moderate abdominal pain; mucus or blood in stool	Severe or persistent abdominal pain, fever, ileus, or peritoneal signs	Life-threatening consequences; immediate intervention required	Death
Transaminitis	3 × ULN	3–5 × ULN	5–20 × ULN	Life-threatening consequences; immediate intervention required	Death
G1 transaminitis with elevated bilirubin (unless Gilbert’s syndrome)			Bilirubin 1–2 × ULN	Bilirubin 3–4 ULN	Death
Elevation in amylase/lipase (no symptoms)	3 × N for both enzymes	3–5 × N	≥5 N		Death
Acute pancreatitis		Asymptomatic lipase and amylase elevation or computed tomography findings indicative of pancreatitis	Symptomatic pain or vomiting and enzyme elevation or computed tomography findings typical for pancreatitis	Radiologic or clinical features of pancreatitis that are life-threatening or hemodynamic instability or any need for urgent intervention	Death

ICI-induced diarrhea or colitis

Differential Diagnosis

When diarrhea occurs in a patient treated with an ICI, an infectious cause should be excluded first. For this, nucleic acid amplification tests for gastrointestinal pathogens should be collected, and a coproculture should be performed. A sample for *Clostridium difficile* will be collected, and a diagnosis of *Giardia *and *Cryptosporidium spp.* infection or *Entamoeba ​​​​​​histolytica* or *Cyclospora/Isospora spp.* infection will be considered. Tests for viruses with intestinal tropism may also be considered [16].

Treatment Options: The Role of the Gastroenterologist

It is ideal for the gastroenterologist to be involved in the treatment of these conditions from the beginning. However, the number of cases and the relatively low severity of most cases mean that grade 1 side effects are managed by an oncologist, with loperamide-type antidiarrheals as the primary treatment. Electrolyte-rebalancing therapy and oral rehydration are also administered. If the intensity of diarrhea remains mild but persists over time, mesalamine or cholestyramine may also be considered [17,18].

Assays to detect lactoferrin and calprotectin are very useful if available in the clinic. If lactoferrin is positive, colonoscopy should be considered as soon as possible (within the first two weeks after symptom onset). If contraindications for colonoscopy are present, at least sigmoidoscopy with biopsy should be performed. Calprotectin is very useful to guide treatment and is ideally interpreted over time (a proposed approach is follow-up for two months, and the intervals between assays should be personalized for every patient). When the calprotectin level returns to normal, the treatment of immune-mediated colitis may be discontinued [16]. It is our firm opinion that these tests can preclude the over-investigation of patients with diarrhea after the treatment with ICIs.

In the case of grade 2 diarrhea/colitis, the intervention of the gastroenterologist and the performance of a colonoscopy, or at least a flexible sigmoidoscopy with biopsy, are mandatory. Depending on the symptoms and the clinical availability of these test methods, upper digestive endoscopy may be required. At this level of toxicity, immunotherapy treatment can be continued, and prednisone/methylprednisolone at 1-2 mg/kg/day is initiated as soon as possible. If no clinical response occurs within two to three days, infliximab (intravenously [IV] at an initial dose of 3 mg/kg at weeks 0 [baseline], 2, and, if needed, 6) can be added to this treatment [19,20]. Alternatively, vedolizumab may be added within two weeks [21].

The duration of treatment with tumor necrosis factor-alpha blockers or integrin blockers is not standardized and will be based on clinical and endoscopic evaluation. Up to three doses have been used in the literature (at weeks 0, 2, and 6), but the last dose may be omitted depending on the patient’s response. The patient must be assessed for tuberculosis before this treatment is administered [16].

In patients who initially present with diarrhea or grade 3 colitis, anti-CTLA-4 should be discontinued, but re-administration of anti-PD-1/PD-L1 may be considered after amelioration. The patient will be treated in the setting of continuous hospitalization (ideally in a gastroenterology clinic), but permanent communication with the treating oncologist is mandatory. Intravenous methylprednisolone (1 to 2 mg/kg/day) will be administered from the beginning, and as in the case of grade 2 toxicity, infliximab or vedolizumab will be added depending on the evaluation. Grade 4 toxicity denotes that ICI therapy will be permanently discontinued [22].

The approach in this situation is similar to that for grade 3 but, of course, includes much more careful monitoring of the patient. In patients who are refractory to infliximab or vedolizumab, oral treatment with tofacitinib, a Janus kinase inhibitor, for 30 days demonstrated very encouraging results [23].

ICI-induced hepatitis

Differential Diagnosis

In patients with elevated transaminases who are receiving ICI therapy, alpha-1-antitrypsin, ferritin, antinuclear antibodies (ANA) titer, mitochondrial antibody, ceruloplasmin, smooth muscle antibody, tissue transglutaminase IgA and IgM, liver-kidney microsome type 1 antibody, tissue transglutaminase IgA, IgG, and thyroid-stimulating hormone will be measured to determine the differential diagnosis [24].

Worsening of preexisting liver damage should be excluded after which the following will be considered: hepatitis virus A, B, or C; cytomegalovirus; Epstein-Barr virus; herpes simplex virus; varicella-zoster virus; and human immunodeficiency virus. Toxic hepatitis caused by acetaminophen, dietary supplements, or alcohol consumption should also be ruled out [16].

Treatment Options: The Role of the Gastroenterologist

The therapeutic approach toward immune-mediated hepatic toxicity depends primarily on bilirubin values. It is crucial to determine whether the increase in bilirubin is in the context of Gilbert’s syndrome. In patients with normal bilirubin values and transaminase levels not higher than three times the normal values, monitoring their trends is sufficient. Assessments of toxicity in any degree begin with an abdominal ultrasound. If the values increase within 24 h, discontinuation of immunotherapy is ideal. For moderate increases in transaminases (three to five times higher than the normal values), the patient will be closely monitored, immunotherapy will be discontinued, and prednisone at a dose of 0.5-1 mg/kg/day will be considered. The workup in all cases consists of not only the levels of total bilirubin and transaminases but also complete blood count, prothrombin time, and coagulation factors [16,25].

The values will be monitored every three to five days, and when they reach grade 1 toxicity again, steroid tapering can be initiated, which is a process that is recommended to last one month. If the transaminases increase again once the corticotherapy doses are decreased, the doses should again be escalated [26].

At values consistent with grade 3 toxicity (five times higher but 20 times lower than the normal values), the patient will be admitted to inpatient care, immunotherapy will be discontinued, and 1-2-mg/kg/day prednisone therapy (or equivalent) will be initiated. Transaminases will be monitored daily, and if no improvement is observed, mycophenolate will be added. Infliximab is contraindicated in cases of liver damage of any degree, and most guidelines advise against using it for immune-mediated hepatitis [27].

At life-threatening toxicity (grade 4) with transaminase values above 20 times higher than the normal values, ICI therapy will never be resumed. The initial treatment also involves corticotherapy alone, which consists of prednisone/methylprednisolone at a dose of 1-2 mg/kg/day for three days. If the transaminase levels do not decrease, mycophenolate will be added. Liver biopsy will be considered if there are no contraindications. If bilirubin is increased by two times the normal values, the grade 3 toxicity treatment regimen will be followed regardless of the transaminase values. In contrast, if bilirubin values are higher than three times the normal values, the toxicity is considered grade 4 and is treated as such. The mycophenolate mofetil dose is 0.5-1 g for 12 h and is administered together with corticosteroid therapy until steroid tapering is needed. When tapering is initiated, mycophenolate treatment should also be discontinued [16,28].

If treatment with corticosteroids and mycophenolate is ineffective, globulin anti-thymocyte administration is possible; the efficacy of which has been demonstrated in at least one case report [29]. A comparison of the recommendations for immune-mediated diarrhea/colitis and hepatitis as presented in the current guidelines (National Comprehensive Cancer Network [NCCN] and European Society for Medical Oncology [ESMO]) was made in Table 2.

**Table 2:** Comparison of the recommendations for immune-mediated diarrhea/colitis and hepatitis as presented in the current guidelines (NCCN and ESMO) [16,24] NCCN, National Comprehensive Cancer Network; ESMO, European Society for Medical Oncology; ICI, immune checkpoint inhibitor; CTLA, cytotoxic T-lymphocyte-associated protein; PD-1, programmed cell death protein-1; PD-L1, programmed cell death protein-1 ligand.

Toxicity/Therapy Guideline	Grade 1	Grade 2	Grade 3	Grade 4
Diarrhea/Colitis NCCN	Holding ICI at oncologist discretion. Loperamide or diphenoxylate + hydration. If persistent, check for infectious etiology. Lactoferrin/calprotectin values (if persistent) → positive → treat as grade 2.	Hold ICIs. Prednisone or equivalent (1 to 2 mg/kg/day). If persistent, consider starting infliximab or vedolizumab (in 2 weeks).	Inpatient care. Anti-CTLA-4 should be discontinued permanently; anti-PD-1/PD-L1 therapy could be resumed if toxicity subsides. Methylprednisolone 1 to 2 mg/kg/day. Monitor for 2 days, if unresponsive, add infliximab or vedolizumab.	Inpatient care. All ICIs should be permanently discontinued. Methylprednisolone 1 to 2 mg/kg/day. Monitor for 2 days, and if unresponsive, add infliximab or vedolizumab.
Diarrhea/Colitis ESMO	ICIs can be continued. Loperamide or any antidiarrheal medication.	Hold immunotherapy. Oral corticosteroids 1 mg/kg or budesonide. If symptoms persist for 3–5 days→ colonoscopy → colitis (lesions visible) → infliximab.	Inpatient care. All ICIs should be permanently discontinued. (Methyl)prednisone 2 mg/kg i.v. → if persistent after 2 to 3 days → infliximab.	Same as grade 3.
Hepatotoxicity (elevated transaminases without high levels of bilirubin) NCCN	3× normal values → consider withholding immunotherapy. Monitor the patient.	3–5× normal values. Hold immunotherapy, and monitor transaminases every 3–5 days. Consider administering prednisone at 0.5–1 mg/kg/day.	5–20× normal values. Hold ICIs. May be admitted to inpatient care. Prednisone at 1 to 2 mg/kg/day → if no improvement, consider mycophenolate mofetil. No infliximab for hepatic toxicity.	20× normal values. Inpatient care + hepatology consultation. Permanently discontinue ICIs. Prednisone/methylprednisolone 1 to 2 mg/kg/day → if no improvement in 3 days, start mycophenolate mofetil.
Hepatotoxicity (elevated transaminases without high levels of bilirubin) ESMO		Hold ICI. Monitor transaminases twice weekly → If no improvement, in 1 week, start 0.5–1 mg/kg (methyl)prednisone.	Discontinue ICIs immediately. (Methyl)prednisolone at 1 to 2 mg/kg/day → monitor for 2 days → if no improvement, start mycophenolate mofetil at 1000 mg three times daily.	Inpatient care. Permanently discontinue ICIs. Methylprednisolone at 2 mg/kg/day, monitor for 2 days → if no improvement, start mycophenolate mofetil at 1000 mg three times daily → if no improvement under double immunosuppression, consider administering anti-thymocyte globulin or tacrolimus.
Hepatotoxicity (high levels of bilirubin) NCCN			1-2× normal values. Hold ICIs. Admission to inpatient care + hepatology consultation. Prednisone/methylprednisolone at 1 to 2 mg/kg/day → if no improvement, consider administering mycophenolate mofetil.	3-4× normal values. Permanently discontinue ICIs. Admission to inpatient care + hepatology consultation. Prednisone/methylprednisolone at 1 to 2 mg/kg/day → if no improvement, consider mycophenolate mofetil.
Hepatotoxicity (high levels of bilirubin) ESMO			Consider inpatient care. Discontinue ICIs. Methylprednisolone at 2 mg/kg/day, monitor for 2 days → if no improvement, start mycophenolate mofetil at 1000 mg three times daily → if no improvement under double immunosuppression, consider administering anti-thymocyte globulin or tacrolimus.	

ICI-induced pancreatitis

Differential Diagnosis

ICI-induced pancreatitis is a clinical entity that requires the intervention of a gastroenterologist from the moment of suspicion. Determination of amylase and lipase levels is not included in the standard assessment of immunotherapy sessions. However, determination of these levels may be required if the patient experiences back pain, diffuse abdominal pain, belching, bloating, or nausea. Even with these symptoms, high values can be present in situations where certain causes of acute pancreatitis, such as irritable bowel syndrome, inflammatory bowel disease, bowel obstruction, gastroparesis, alcohol, medication, or diabetes mellitus, are absent. To make this differential diagnosis, it is essential to obtain a detailed history from the patient [16,24].

Treatment Options: The Role of the Gastroenterologist

If the gastroenterologist highly suspects acute pancreatitis, computed tomography (CT) with contrast will be performed. The classic diagnosis of acute pancreatitis involves intense abdominal pain and vomiting accompanied by high levels of amylase and lipase. Magnetic resonance cholangiopancreatography will be considered if the clinical suspicion is high, but CT would not reveal any changes. In cases where immune-induced pancreatitis is highly suspected, the patient will be admitted to a gastroenterology department, the pain will be treated, and the patient will be kept hydrated and monitored for hydroelectrolytic imbalances. If the suspicion based on imaging is confirmed or if the condition worsens clinically or biologically, prednisone/methylprednisolone at 0.5-1 mg/kg/day is initiated. Obviously, from grade 2 toxicity onward, immunotherapy will no longer be administered. Evidence that monitoring the patient via CT is necessary during treatment is lacking. The decision to taper corticotherapy will be based primarily on the patient’s clinic combined with the patient’s serum lipase and amylase values. Generally, this condition will be treated until the toxicity decreases to grade 1. If the patient exhibits hemodynamic instability or if the radiological changes are considered life-threatening, the toxicity will be considered grade 4, and immunotherapy will be permanently discontinued. Of course, these decisions remain at the discretion of the multidisciplinary team.

Even if the episode is successfully treated, the patient will be monitored over the long term for exocrine pancreatic insufficiency and will be treated appropriately if this occurs. It should be considered that such an episode can induce the development of diabetes mellitus, and patients should be monitored for this reason [16,24].

Several aspects of the gastroenterologist’s perspective require more detailed research. First, the course of the mucosal lesions is unclear. It seems that the distribution of the lesions is patchy, but we do not yet know how they evolve. Staging according to endoscopic and pathology features is also unavailable, and the use of criteria for inflammatory bowel disease has not been demonstrated to be useful for guiding the treatment of immune-induced colitis. Although being the primary treatment for these side effects, corticosteroid therapy does not have a standard route of administration (it is not known whether oral or IV administration is superior, and there is no preferred agent). Corticosteroid withdrawal time and tapering of doses are also not standardized [29].

These aspects derive from the finding that, especially in the beginning, most side effects are managed by oncologists, but they will all become more frequent, as the use of ICIs is already widespread for many solid tumor types. Awareness of these conditions is a priority in the gastroenterology community. Prospective randomized trials are needed to develop new treatment guidelines for this toxicity. Furthermore, better outcomes in treating immune-induced toxicity will emerge with the accumulation of experience by gastroenterologists [29].

## Conclusions

ICIs are becoming an increasingly important component in systemic oncological treatment, and they can be administered in almost all types of solid tumors. As their use increases, the frequency of immune-mediated adverse reactions, including gastroenterological events, will also increase. Therefore, the gastroenterology community should know of the existence of this new disease and should be able to collaborate as a multidisciplinary team to treat these patients. Moreover, it is imperative that gastroenterologists be involved in research that will provide a new guideline for the management of these toxicities. This review aims not only to inform about the latest treatment trends but also to mobilize the community to actively improve the care of patients with these side effects by bettering the research in general.
